# Neurodevelopment in Down syndrome: Concordance in humans and models

**DOI:** 10.3389/fncel.2022.941855

**Published:** 2022-07-15

**Authors:** Jenny A. Klein, Tarik F. Haydar

**Affiliations:** ^1^Graduate Program for Neuroscience, Boston University, Boston, MA, United States; ^2^Children’s National Hospital, Center for Neuroscience Research, Washington, DC, United States; ^3^Departments of Pediatrics, Physiology and Pharmacology, School of Medicine and Health Sciences, George Washington University, Washington, DC, United States

**Keywords:** down syndrome, neurodevelopment, human tissue, mouse model, iPSCs

## Abstract

Great strides have been made over the past 30 years in understanding the neurodevelopmental changes underlying the intellectual disability (ID) in Down syndrome (DS). Detailed studies of human tissue coupled with findings from rodent and induced pluripotent stem cells (iPSCs) model systems have uncovered the changes in neurogenesis, synaptic connectivity, and myelination that drive the anatomical and physiological changes resulting in the disability. However, there remain significant conflicting data between human studies and the models. To fully understand the development of ID in DS, these inconsistencies need to be reconciled. Here, we review the well documented neurodevelopmental phenotypes found in individuals with DS and examine the degree to which widely used models recapitulate these phenotypes. Resolving these areas of discord will further research on the molecular underpinnings and identify potential treatments to improve the independence and quality of life of people with DS.

## Introduction

It has been nearly 200 years since John Langdon down first described Down syndrome (DS) and 63 years since Jerome Lejeune identified triplication of chromosome 21 (HSA21) as its cause (Down, 1866; Lejeune et al., 1959). Since then, much has been learned about the molecular features of DS, its effects on human development throughout the lifespan, and the many capabilities and joys that people with DS bring to their families and communities. Research efforts to understand the causes and effects of trisomy 21 come from hundreds of laboratories across the planet; their main goal is to uncover the biological events that most impact the lives of people with DS so that approaches can be developed to improve their quality of life and independence. While several of the effects of trisomy 21 are currently medically managed, such as cardiac and gastrointestinal tract defects, the most penetrant feature of DS – the intellectual disability (ID) – has thus far not been sufficiently understood to develop successful therapies. This is partly due to the complexity of the human brain and its inaccessibility to longitudinal, invasive studies, but is also due to the fact that each person with DS is an individual with their own background genetics, environmental exposure, and expression of DS features. While the past decades of DS research have yielded significant advances, there is now a need to assess those features of the disorder that remain elusive and plan the next phase of investigation into how the human brain responds to HSA21 triplication.

In this review, we will frame the effort to understand the ID in people with DS, first by describing what is known from human studies and then by examining the different model systems that are used to study brain development and function in DS. In doing so, we will emphasize both the areas of development that share strong concordance between the human record and the model systems, those areas of discordance between the models and people with DS, and possible approaches to address these divides between the clinical features that appear across the lifespan and the molecular mechanisms that cause them.

## Central nervous system alterations in humans with Down syndrome

Today, DS is the most common genetic form of ID with a prevalence of 1 in 700 live births in the United States (Mai et al., 2019). While some aspects of the ID can be modulated with interventional therapies, there are currently no treatments aimed at the molecular mechanisms that cause ID, and so it therefore remains a significant factor limiting the independence and quality of life of people with DS. The ID in DS ranges from moderate to severe as measured by intelligence quotient (IQ 10–70) with a median IQ of 40 (Guéant et al., 2005). It presents as a range of impairments including predominant deficits in executive functioning and memory (Vicari et al., 2005, 2000; Lanfranchi et al., 2010; Costanzo et al., 2013; Grieco et al., 2015). The impairments in executive functioning include deficits in attention, inhibition, planning and organization, set-shifting, and self-monitoring, all of which are critical categories of intellectual function needed for independent living (Cornish et al., 2007; Abbeduto et al., 2008; Trezise et al., 2008; Lanfranchi et al., 2010; Breckenridge et al., 2013; Costanzo et al., 2013). The memory impairment includes deficits in both short-term and long-term declarative memory, with procedural memory comparable to typically developing peers (Carlesimo et al., 1997; Pennington et al., 2003; Vicari et al., 2005, 2000; Godfrey and Lee, 2018). Together, these impairments in distinct cognitive areas culminate in the ID displayed in individuals with DS. If the physical underpinnings of the ID in DS could be identified and understood, the development of pharmacological treatments to help increase the quality of life for individuals with DS would be possible.

### Anatomical changes

Although it is still not currently possible to identify the exact causes of the ID in DS, the scientific record clearly points to structural changes that generate functional alterations in the brains of people with DS. Post-mortem and magnetic resonance imaging (MRI) studies consistently show that the overall size of the brain is decreased compared to age-matched controls. In particular, the cerebral cortical hemispheres, cerebellum, and hippocampal formations are significantly reduced in size (Wisniewski, 1990; Golden and Hyman, 1994; Kesslak et al., 1994; Raz et al., 1995; Pinter et al., 2001; Tarui et al., 2020; McCann et al., 2021). These structural changes correlate with some of the specific deficits noted in cognitive function in DS. For example, the brain structures that are reduced in size in DS are the structures that are responsible for executive function (frontal lobe, parietal lobe, and cerebellum) (Nowrangi et al., 2014) and memory – specifically declarative memory (hippocampus) (Scoville and Milner, 1957; Squire and Zolamorgan, 1991; Bliss and Collingridge, 1993).

The neuroanatomical differences in size are present starting at birth; changes in volume were identified in multiple cohorts consisting of individuals ranging from 0 months to 5 years of age (Schmidt-Sidor et al., 1990; McCann et al., 2021) implicating onset during fetal development. Indeed, as early as 23 weeks gestation, volumetric changes are apparent in developing fetuses with DS (Golden and Hyman, 1994) while no changes were observed in fetuses 15–22 weeks of gestation (Schmidt-Sidor et al., 1990). Analysis of fetal brain MRIs show that the cortical plate, subcortical parenchyma, and cerebellar hemispheres have significantly decreased growth trajectories in fetuses with DS compared to typically developing controls beginning at 28 weeks of gestation (Tarui et al., 2020). Additionally, it appears that these volumetric differences become more disparate with age. Though present at birth, distinct size differences were more apparent after 3–5 months of age (Schmidt-Sidor et al., 1990) and in a detailed MRI study, the youngest cohort (0–5 years) showed a smaller effect size compared to older cohorts (5–10, 10–15, and 15–20 years) indicating more similarly between the individuals with DS and controls at the younger age (McCann et al., 2021).

Together, brain measurement studies consistently indicate differences by the early second trimester of gestation, a developmental phase when critical processes such as cellular maturation, synapse formation, and dendrite growth occur. Importantly, the fact that specific areas of the brain are more affected than others indicates that neurodevelopment may be regionally altered during prenatal growth, a particularly interesting and difficult problem to address since it is still unknown how regional differences develop in typically developing brains. Compounding this, as you will see below, current model systems are unable to approximate the complex spatial and temporal development of the human brain, so this major facet of DS brain development (regional differences in growth) continues to remain a mystery. Nevertheless, the basic developmental processes at work in the brain, such as neurogenesis, gliogenesis and synapse growth, are necessary in all developing areas. Thus, differences in these processes that are common between people with DS and between brain regions may provide an access point for the development of interventional strategies.

### Neuronal correlates of anatomical changes

The observed differential growth rate leading to structural changes may in turn be driven by changes in neurogenesis. Reductions in the number of proliferating neural progenitor cells (NPCs) in the dentate gyrus, germinal matrix of the lateral ventricle, and third ventricle have all been observed in tissue derived from fetuses diagnosed with DS (17–21 weeks gestation) prior to the initial observations of volumetric changes (Contestabile et al., 2007; Stagni et al., 2020). This reduced proliferation appears to result in a smaller NPC pool as fewer SOX2+ cells have been reported in the outer subventricular zone (oSVZ) in the developing cerebral cortex in fetuses with DS during mid-gestation (18–24 weeks) potentially limiting the production of neurons and glia (Baburamani et al., 2020). General hypocellularity has consistently been identified in multiple structures at numerous points of development. At 19 weeks gestation, a significant decrease in total cells was found the forebrain (Larsen et al., 2008). During the same time period (17–21 weeks gestation) fewer neurons were found in the hippocampus (Guidi et al., 2008), cerebellum (Guidi et al., 2011), and multiple thalamic nuclei (Stagni et al., 2020). However, other studies have not identified any change in cellular density in the superior temporal neocortex prior to 23 weeks gestation and instead report DS related hypocellularity only appearing later in development (Schmidt-Sidor et al., 1990; Golden and Hyman, 1994). The hypocellularity persists with fewer total neurons being identified in the superior temporal gyrus in adolescents and adults with DS (15–35 years) (Ross et al., 1984; Giffin-Rao et al., 2020). While the timing may be disputed, the consensus is that by late gestation there are fewer cells in some areas of the brain in individuals with DS and this change persists into adulthood.

The decrease in production of neurons is just the initial neurodevelopmental deficit identified in the gray matter in DS. As volumetric differences between individuals with DS and control individuals continue to diverge postnatally after neurogenesis ends, other neurodevelopmental processes must also be perturbed, including dendrite arborization and synapse formation (de Graaf-Peters and Hadders-Algra, 2006). Initially, in infants aged 0–6 months, the total dendritic length was longer in samples from individuals with DS compared to controls. However, as development continued this difference reversed, with dendritic length becoming significantly shorter in samples from individuals with DS and persisting at least into childhood (7 years) (Becker et al., 1986). Following a similar pattern of exacerbating changes, another study showed that fetuses (14–40 weeks gestation) with DS had similar dendritic spine counts compared to controls. However, postnatally, infants (0–12 months) presented with shorter dendrites and a decreased number of spines in the visual cortex (Takashima et al., 1981). These dendritic and synaptic changes appear to persist into adulthood as a decreased number of dendritic spines on pyramidal neurons were found both in children and young adults (3–23 years) (Suetsugu and Mehraein, 1980) and middle aged adults (47–55 years) compared to age matched controls (Ferrer and Gullotta, 1990).

Finally, the subtype specificity of the neurons altered in DS is currently unknown. Of particular interest is the change in the ratio of excitatory to inhibitory neurons as this would have a large effect on circuit activity and level of excitability. Broadly, one study has identified a decreased number of aspinous stellate interneurons in young adults with DS (Ross et al., 1984). However, another study analyzing a similar age group and area found no difference in parvalbumin or calretinin positive interneurons (Giffin-Rao et al., 2020). A third study also found no difference in the density of interneurons in thalamic nuclei of fetuses (17–21 weeks gestation) with DS compared to those without (Stagni et al., 2020). However, because this same study identified a significant decrease in density of excitatory neurons, the proportion of interneurons to excitatory neurons was significantly increased in fetuses with DS. A similar finding was made in the cortical plate in the developing fusiform gyrus and inferior temple gyrus (17–21 weeks gestation). There was no significant difference in the number of calretinin positive interneurons between fetuses with DS and those without, but due to a decrease in number of calretinin negative cells, the proportion of calretinin positive interneurons to the total number of neurons was higher in the samples from individuals with DS (Guidi et al., 2018). This finding of increased calretinin positive interneuron proportion without increases in interneuron number, was repeated in the entorhinal cortex and hippocampus (Guidi et al., 2018). A recent study supported this finding in tissue from the prefrontal cortex of adolescents and young adults with DS (13–32 years). Again, no difference in percentage of interneurons (measured by GAD67 protein expression) was identified, but the proportion of interneurons to total neurons was significantly increased in DS (Palmer et al., 2021).

The last study also used single nucleus RNA-seq to examine neuronal composition. They identified six clusters of inhibitory neurons that expressed *ADARB2*, indicating they arose from the CGE, and four clusters that expressed *LHX6*, indicating they arose from the MGE. Interestingly, the fraction of neurons that expressed *ADARB2* was significantly increased in the samples from individuals with DS, while the fraction of neurons that expressed *LHX6* was significantly decreased. The analysis was also performed with the addition of an older cohort (39–65 years). Again, an increase in the fraction of *ADARB2* expressing interneurons was identified, but no difference in *LHX6* interneurons was found. The opposing changes based on developmental origin may explain the conflicting results identifying an increase or decrease in interneurons in different studies. As the presence of this phenotype would greatly affect neuronal circuitry and development of ID in DS, further study to reconcile the conflicting data is essential.

### Myelination and oligodendrocyte perturbations

In addition to changes in neuronal composition, numerous studies have identified perturbations in glia in DS, particularly in oligodendrocytes (OLs). Delayed myelination was identified in children with DS (2 months–6 years), with an average delay of 12 months compared to typically developing controls (Wisniewski and Schmidt-Sidor, 1989). The delay predominantly affects associated and intercortical fibers in the fronto-temporal lobes, tracts that typically display late initiation of myelination (Wisniewski and Schmidt-Sidor, 1989). Myelination is also delayed in the hippocampus in DS (Ábrahám et al., 2012). In addition to the developmental delay, the density of the final myelinated axons is decreased in DS (Ábrahám et al., 2012; Olmos-Serrano et al., 2016a). As myelin acts as an electrical insulator to increase the conduction velocity of action potentials, decreases in myelination likely affect neuronal communication in DS and may contribute to the ID. The typical grid-like matrix found in myelinated fiber in the cerebral cortex is also disrupted and lower levels of myelin associated proteins, myelin basic protein (MBP) and myelin associated glycoprotein (MAG) have also been detected (Olmos-Serrano et al., 2016a). In addition, lower levels of 2′,3′-cyclic nucleotide-3′-phosphodiesterase (CNP), an OL specific protein, have also been detected in the front and temporal lobes of tissue samples from DS versus controls (Vlkolinský et al., 2001). Finally, in a separate study, analysis of RNA isolated from cortical samples across the developmental lifespan (14 weeks gestation–40 years), identified the downregulation of a module enriched for genes expressed in the OL lineage (Olmos-Serrano et al., 2016a). This indicates that there may be intrinsic changes in OL production and differentiation which may result in the decrease in white matter production that has been observed in postnatal and adult samples.

### Astrocytic and microglial changes

In addition to changes in myelination and OLs, differences in the other glial cells in the CNS, astrocytes and microglia, have also been described in the brains of people with DS. In addition to their homeostatic and immune functions in the adult brain, both astrocytes and microglia have long been shown to play important roles in synaptic development and modulation of neuronal circuitry during development (Reemst et al., 2016). Changes in their number or functional ability during this early developmental period in DS may influence the changes in synaptic connectivity described above. Understanding the full interplay of neurons and glia in shaping final neuronal circuitry is important for understanding the cellular underpinnings of the ID in DS.

An increased number of astrocytes has been observed in the frontal lobe (Zdaniuk et al., 2011), fusiform gyrus, and inferior temporal gyrus (Guidi et al., 2018) of developing fetuses with DS compared to those without (17–21 weeks). A separate study also found an increase in immunoreactivity of GFAP and the astrocytes showed a more activated morphology compared to control tissue (Chen et al., 2014). The increase in astrogliogenesis concurrent with a reduction in neurogenesis is thought to be indicative of a gliogenic shift in development though the consequences of this shift are still unknown.

Finally, changes in the third type of glia, microglia, have also been identified in DS. While microglia are not of neuroectoderm origin but rather derive from myeloid progenitors in the yolk sac, they migrate into the developing CNS starting at 4.5–5.5 gestational weeks in humans and play an important role in multiple neurodevelopmental processes (Ginhoux et al., 2010; Verney et al., 2010; Reemst et al., 2016). Therefore, in the context of perturbed neurodevelopment in DS, they are important to consider. There is an increase in the number of ramified microglia in developing fetuses with DS compared to controls (18–22 weeks) (Wierzba-Bobrowicz et al., 1999). A separate study also identified the upregulation of microglia related genes in hippocampal samples derived from adolescents and adults with DS (13–39 years) as well as an increase in expression of IBA1, a marker of activated microglia. In an older cohort (36–67 years), deficits in microglia morphology including decreased ramification and increased cell body size were identified in samples from individuals with DS (Pinto et al., 2020). Similar to the increase in astrogliogenesis, the consequences of this microglia dysregulation on neurodevelopment are as of yet unknown.

### Cellular changes in the spinal cord

While the majority of studies have focused heavily on identifying perturbations in brain development in DS, there are also alterations in the cellular composition of the spinal cord that are thought to contribute to the motor deficits found in DS (Morris et al., 1982; Palisano et al., 2001). At present, there is only one study examining cellular changes in the spinal cord that may underlie this deficit. A decrease in choline acetyltransferase (ChAT)-positive motor neurons was identified in cervical sections of the spinal cord from older adults with DS (58–70 years) (Watson-Scales et al., 2018). However, motor deficits are present at birth indicating that changes in cellular circuitry of the spinal cord likely precede adulthood. Further work will be needed to quantify changes in neuronal subtypes and glia to further understand changes in this area of the CNS in DS.

### Importance of human studies in Down syndrome and future work

**FIGURE 1:**
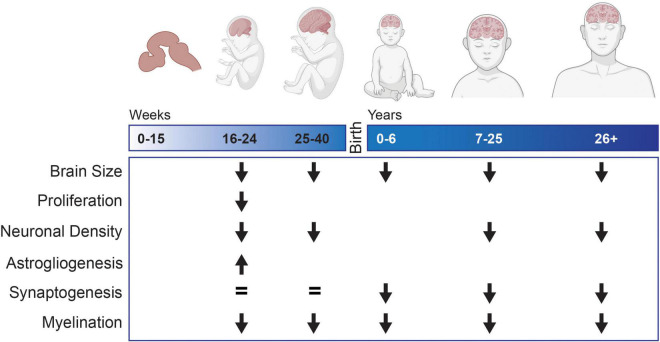
Neurodevelopmental changes observed in tissue samples or MRI from individuals with Down syndrome compared to typically developing controls. Blank spaces indicate that no data exists for that developmental time period.

The changes observed directly in human samples from individuals with DS are the most reliable information about different neurodevelopmental phenotypes that may be underlying the ID. These known phenotypes (Figure 1) should be considered the benchmark that model systems need to recapitulate in order to be considered a model of DS. It should be noted that much of what we know of neurodevelopment in DS during late gestation and childhood is derived from tissue samples from individuals that died prematurely. These individuals were likely more severely affected by the syndrome and the developmental changes noted during these time points may differ in either magnitude or type compared to less affected individuals. The genetic correlates of the variability of phenotypic presentation in DS are still unknown and is an area that requires further research for a more complete understanding of the syndrome. Animal models that live into adulthood and cellular models derived from healthy people with DS may not fully capture some of the neurodevelopmental changes observed in the most affected individuals. However, until the field fully understands how neurodevelopment varies along the spectrum of the disorder, the phenotypes observed from tissue studies from individuals with DS should still be considered the gold standard, especially if the phenotype is consistently identified. If a model is not able to recapitulate these known phenotypes, consideration should be taken as to whether it is a reliable model and if the information learned from it is directly relevant to understanding changes in neurodevelopment in individuals with DS.

Identification of reliable model systems is essential to understand the underlying mechanisms of the ID in DS due to inherent limitations of fixed tissue studies and longitudinal analyses to directly identify causative cellular processes are not possible in human subjects. The majority of the changes observed in human tissue studies are only a snapshot of the developmental processes at that specific time point; thus, the changes leading up to the observed differences and their effects can be inferred but cannot directly be known. Because of these challenges, the field has turned to models to try to understand the cellular and molecular mechanisms underlying the ID.

## Modeling neurodevelopment with animal models

Mouse models of DS have been a mainstay of translational research for decades. The first mouse model of DS was the Trisomy 16 (Ts16) mouse which harbored triplication of mouse chromosome 16 (MMU16), containing a large region of genes syntenic to those on HSA21 as well as many genes that are not represented on HSA21 (Reeves et al., 1986). While this mouse partially modeled the genetics of DS, the trisomy was perinatally lethal, so experiments were limited to embryonic periods, preventing understanding of postnatal features of DS. As technology improved, subsequent generations of mouse models of DS have been generated, resulting in models that survive postnatally, and which are more genetically comparable to humans with DS. These rodent models have been an extremely valuable system to study the cellular and molecular underpinnings of neurodevelopmental changes and have allowed experimental manipulations that are not possible in limited human tissue. The rapid increase in scientific publications on DS (1,847 articles published in 2020 compared to 624 published in 1990) is due to the availability of these models and has illuminated many of the mechanisms driving the neuronal phenotypes of DS. However, the understanding gained from these models is only as good as their ability to accurately replicate the neurodevelopmental changes observed in people with DS and so they need to be assessed carefully before being used to draw conclusions about the human syndrome. There are currently more than a dozen mouse models, but here we cover a few of the more widely used and most recent mouse models to assess how consistently they model DS related phenotypes and whether we can extrapolate the underlying mouse cellular mechanisms back into humans.

### Ts65Dn

Historically the most widely used, and therefore best characterized mouse model of DS, is the Ts65Dn mouse. Unlike the Ts16 mouse model, trisomic Ts65Dn survive to adulthood enabling postnatal studies for the first time in a mouse model (Davisson et al., 1993). Unlike the triplication of a full MMU16, Ts65Dn is the result of a translocation of the distal region of MMU16 onto the centromeric region of mouse chromosome 17 (MMU17). Therefore, like the majority of individuals with DS, Ts65Dn has an extra freely segregating marker chromosome that carries ∼119 genes triplicated from MMU16 (Duchon et al., 2011). However, due to the nature of the translocation, 60 of the non-syntenic genes on MMU17 are also triplicated in Ts65Dn and their impact on the observed phenotypes is unknown. Behaviorally, Ts65Dn appears to capture many of the features of DS including developmental delays and learning and memory deficits (Reeves et al., 1995; Olmos-Serrano et al., 2016b; Shaw et al., 2020). Therefore, Ts65Dn has been considered a good model to understand the cellular underpinnings that connect the genetics of DS to the ID.

#### Recapitulation of neurodevelopmental phenotypes

The Ts65Dn mouse model recapitulates some of the anatomical and cellular phenotypes found in human studies that are thought to underlie the development of the ID. Similar to human studies, neuronal hypocellularity has been detected in multiple structures including the cortex, cerebellum, and hippocampus in postnatal trisomic animals (Insausti et al., 1998; Baxter et al., 2000; Olson et al., 2004; Roper et al., 2006; Chakrabarti et al., 2010; Aziz et al., 2018). However, unlike humans, this hypocellularity does not appear to be driving persistent volumetric changes in these structures, as size deficits detected prenatally (Chakrabarti et al., 2007) do not persist postnatally except in the cerebellum (Baxter et al., 2000; Olson et al., 2004). However, these recapitulated phenotypes appear to be subject to phenotypic drift. In a recent comparison of cohorts of Ts65Dn animals spanning from 2010 to 2019, neither decreases in neuronal density in the cerebellum or hippocampus nor embryonic cortical size differences were measured (Shaw et al., 2020). The cause of this drift is unknown and currently limits the utility of continuing to use the model to study these phenotypes.

Similar to humans, the neuronal hypocellularity in Ts65Dn is thought to be driven by changes in NPC proliferation during development. Increases in the cell cycle length of NPCs in the germinal zone of both the dorsal telencephalon and hippocampus have been found, resulting in slower proliferation and decreasing the expansion of the progenitor pool (Chakrabarti et al., 2007). In concert, the number of actively proliferating cells in the germinal zone of the dorsal telencephalon is decreased (Aziz et al., 2018; Shaw et al., 2020) and this deficit persists postnatally, with fewer proliferating cells being found in the hippocampus (Contestabile et al., 2007). Contrary to the existing human data, these proliferation changes appear to be region specific as an increase in number of actively proliferating cells was observed in the embryonic medial ganglionic eminence, the site of interneuron production (Chakrabarti et al., 2010; Aziz et al., 2018).

Finally, another phenotype consistently observed in tissue samples from individuals with DS is a decrease in neurite arborization and synaptic density. It is thought that this may contribute to the volumetric changes observed in individuals with DS in addition to affecting circuit formation and neuronal communication. Though Ts65Dn fails to capture the majority of the volumetric changes observed in humans, they do appear to recapitulate the synaptic changes as decreases in synaptic density have been identified in both the hippocampus and the neocortex (Belichenko et al., 2004; Chakrabarti et al., 2007). The broad availability of tissue from trisomic Ts65Dn animals has also allowed a more detailed examination of synapses than is currently possible with the limited human tissue samples. Close examination has found that both pre-synaptic boutons and spines are enlarged and the physical distribution of afferent inputs is changed compared to their euploid controls (Belichenko et al., 2004). Additionally, the length of symmetric synapses appears to be significantly longer in the Ts65Dn hippocampus indicating an alteration of structure of typically inhibitory connections. Finally, a significant increase in the number of inhibitory synapses without any changes in excitatory synapses identified (Belichenko et al., 2009). This ability to examine synaptic changes in detail exemplifies the importance of having a model of DS that is experimentally malleable to study the cellular and molecular underpinnings of phenotypes observed in individuals with DS.

While Ts65Dn do not capture all the well described phenotypes identified in individuals with DS, they recapitulate enough salient phenotypes to be used as a model to uncover their underlying mechanisms. However, as the volumetric changes and hypocellularity are some of the most consistent phenotypes described in individuals with DS, the disappearance of them in various cohorts of Ts65Dn currently calls into question their utility as a model to capture key neurodevelopmental aspects of DS. Nevertheless, there is still a broad array of data produced from Ts65Dn that should not be discounted with the current concerns regarding the stability of the model.

#### Using Ts65Dn to uncover cellular underpinnings of phenotypes

A major utility of mouse models of DS is the ability to first recapitulate phenotypes observed in tissue and then identify underlying cellular and molecular mechanisms driving them in a way that is impossible to study in humans, like the structural changes in synapses. A major area of interest in DS research is the nature of the inhibitory and excitatory neuronal balance. Changes in the ratio of these will greatly affect circuit formation and functionality. Results from human studies have been contradictory as detailed in the previous section and Ts65Dn has been used extensively as a model to attempt to understand the nature of these neuronal changes in a more experimentally tractable setting. In young Ts65Dn from postnatal day 8–30, a significant increase in the number of parvalbumin and somatostatin positive interneurons has been measured in the sensorimotor cortex and the hippocampus, though no difference in calretinin positive neurons was identified, contrary to previous data in humans (Chakrabarti et al., 2010). In concordance with human data, though in contrast to the previous work, another study found that there was an increase in the density of calretinin positive interneurons in the Ts65Dn neocortex and no overall increase in parvalbumin interneurons though the distribution of both interneurons types was significantly altered throughout the layers of the cortex (Pérez-Cremades et al., 2010). A third study also assessed the interneuron content of the hippocampus and again identified a significant increase in calretinin positive neurons with no change in parvalbumin positive neurons (Hernandez-Gonzalez et al., 2015). Though discordant on subtype details, this data generally shows that, in Ts65Dn, there is an increase in the density of inhibitory interneurons which supports the human data showing an increase in density compared to all neurons. As this ratio is a key cellular phenotype that would contribute to the ID, obtaining a complete and coherent understanding of changes in interneuron production in both mouse models and human data is important.

The rationale underlying the keen interest in clarifying the interneuron phenotype both in models and in individuals with DS is the functional role they play in controlling neuronal output and communication. The functionality of neurons is something that is impossible to study in human tissue and is an important area of work with mouse models in general. A seminal study in the field found failure of induction of long-term potentiation (LTP) in the Ts65Dn hippocampus due to excessive GABA-mediated inhibition and a wide array of following studies have confirmed this finding (Siarey et al., 1997, 1999; Kleschevnikov et al., 2004; Costa and Grybko, 2005; Fernandez et al., 2007). This over inhibition is thought to be mediated by either the increase in number of interneurons or the increase in synaptic contacts they make in the hippocampus. As LTP is the mechanism behind hippocampal based declarative memory, deficits in the function are thought to be key to understanding the ID in DS and showcases the value that a model of DS can have on understanding the underpinnings of human phenotypes. However, translating these findings back into humans in the form of pharmaceutical interventions has yet to prove successful (Hart et al., 2017). Whether this is due to the physiological gap between human and mice or is more reflective of issues specific to the Ts65Dn mouse model is unclear.

#### Glial changes in Ts65Dn

Human studies have identified changes in all types of glia in addition to the neuronal changes found in the CNS. Importantly, Ts65Dn has historically recapitulated these glial changes and has provided insight into the mechanisms driving them in people. Trisomic Ts65Dn display a similar white matter phenotype to humans with thinner myelin sheaths and decreased amounts of MAG and MBP in the corpus callosum (Olmos-Serrano et al., 2016b). Analysis of Ts65Dn provides an explanation for this decreased myelin *via* a maturation deficit of the OL precursor cells (OPCs). A significantly lower percentage of OPCs mature to CC1+ OLs and more remain as NG2+ immature OPCs in Ts65Dn. This defect appears to be cell autonomous as purified trisomic OPCs from Ts65Dn differentiate into fewer mature MBP+ cells in culture compared to OPCs isolated from control euploid mice (Olmos-Serrano et al., 2016b). Along with suggesting a potential mechanism behind the myelin deficit, Ts65Dn also allows us to observe the potential functional consequence of this decrease myelin in humans. In the mice, action potential transmission is slower along myelinated axons, perhaps contributing to the ID in DS. However, similar to the phenotypic drift resulting in the disappearance of volumetric and density changes in Ts65Dn, examination of several cohorts of Ts65Dn has identified the presence or absence of the phenotype in no discernible pattern. This variability makes further study of this important phenotype difficult in this mouse model as well.

Opposed to the decrease in white matter, human studies have consistently identified an increase in astrocytes as the expense of neuronal generation. This astroglia shift has also been observed in Ts65Dn with an increased percentage of cells showing astroglial morphology in the hippocampus (Contestabile et al., 2007). Additionally, an increase in astrocytes as identified by S100β staining has been found in both the cortex and the hippocampus (Illouz et al., 2019). As this increase appears consistent in the human data, it is worth further study in mouse models as the functional consequence of the increase in astrocytes on neurodevelopment and circuit formation is unclear. There have been few studies of microglia in humans and the results are inconsistent but suggest increased activation, a phenotype that Ts65Dn also appears to recapitulate (Illouz et al., 2019).

#### Spinal cord

Currently, the only described cellular changes in the spinal cord in humans with DS is the reduction in motor neurons. A decrease in ChAT+ motor neurons has also been identified in Ts65Dn mice, but only in aged mice at 10–11 months of age. There was no difference in motor neuron number at postnatal day 60 indicating that in mice, this decrease in motor neurons may be a neurodegenerative phenotype rather than a developmental one (Aziz et al., 2019). While there is only one phenotype specifically described in human spinal cord, other changes in neuronal and glial populations that have been described in the brains of both individuals with DS and Ts65Dn have also been found in the spinal cord. These include a significant increase in the number of calretinin positive interneurons and changes in maturation status of the oligodendrocyte lineage (Aziz et al., 2019). These findings illustrate another characteristic of a good model of DS, hypothesis generation. In the future, it would be useful to example human tissue samples from individuals with DS to specifically look at changes in interneuron and myelin based on the findings in this model.

#### Ts65Dn conclusions

Generally, Ts65Dn recapitulates the aneuploidy, the triplication of genetic material, and learning and memory deficits that are seen in DS and has long been considered a good model of the syndrome. However, many of the phenotypes that have been described in Ts65Dn have recently been found to be susceptible to phenotypic drift and are variable between cohorts (Shaw et al., 2020). This calls into question the current utility of the model to study the molecular and cellular underpinnings of DS. Still, as Ts65Dn has a long history of study, the previous findings are useful for providing a road map to understand the etiology of the ID in DS. As technology advances and newer animal models of DS are generated, the findings from Ts65Dn that recapitulate changes seen in humans can be used as an experimental guide in the newer models.

### Dp1Tyb/Dp(16)1Yey

In addition to the recent issues plaguing Ts65Dn, there have been long held questions about the affect the seventy non-syntenic genes triplicated on MMU17 have on the generation of observed phenotypes. While Ts65Dn represents an early iteration of modeling DS in mice, Dp1Tyb and Dp(16)1Yey represent a second generation of genetic modeling. Here, targeted editing *via* Cre/*loxP* mediated recombination has resulted in the duplication of the entire region on MMU16 syntenic to HSA21, creating one chromosome carrying a duplication of ∼113 genes (Li et al., 2007; Lana-Elola et al., 2016). This chromosome carrying the duplication paired with a non-edited MMU16 results in the triplication of the genes syntenic to HSA21 and eliminates the genetic confounds of the non-syntenic genes in Ts65Dn. These mouse models have quickly become popular models of DS and replicate some of the same developmental and learning and memory deficits as individuals with DS (Goodliffe et al., 2016; Pinto et al., 2020; Lana-Elola et al., 2021).

Unlike Ts65Dn, examination of Dp(16)1Yey failed to find any of the neurodevelopmental phenotypes that have been well described in individuals with DS. No change in brain volume, NPC proliferation, neurogenesis, or neuronal density was identified in the developing forebrain (Goodliffe et al., 2016) nor any changes in cerebellar anatomy in Dp1Tyb (Watson-Scales et al., 2018). A change in interneuron populations have been identified, but unlike the increases found in Ts65Dn and likely in humans, a decrease in parvalbumin and somatostatin interneurons was found at postnatal day 15 in the cortex (Goodliffe et al., 2016).

Although the Dp1Tyb/Dp(16)1Yey models do not show the major changes in neurodevelopment that are thought to underlie the ID in DS, they do recapitulate some of the phenotypes seen both in humans and in Ts65Dn. A decrease in spine density was found in the trisomic Dp(16)1Yey mice that is thought to be due to overactivated microglia in the trisomic animals (Pinto et al., 2020), phenotypes that are in agreement with the human data. Additionally, Dp1Tyb shows the same decrease in motor neurons in the spinal cord at six month of age that has been observed in aged Ts65Dn and humans (Watson-Scales et al., 2018).

While not as extensively studied as the Ts65Dn model, the collected data do not suggest that Dp1Tyb/Dp(16)1Yey is a good model of the neurodevelopmental changes that are thought to underlie the ID in DS. They do recapitulate other phenotypes seen in DS such as cardiovascular abnormalities, skeletal development, and motor function (Li et al., 2007; Lana-Elola et al., 2016, 2021) and thus could be used as appropriate models to study these changes in DS. Even though the model does show learning and memory deficits, they may be a result of divergent neurological changes that do not map with the changes that are thought to underlie the disability in DS. It is also possible that deeper study would show similar changes in white matter or neurological function that may recapitulate changes seen in people with DS, but until there is a better understanding of the model, it should be used with caution to draw conclusions about the cellular underpinnings of the ID in DS.

### Next generation humanized rodent models

While the majority of mouse models of DS, including those described here, utilize the synteny of MMU16 to HSA21 to triplicate relevant DS genes, the latest generation of rodent models of DS have taken a different approach and introduced a stable HSA21 to create chimeric trisomic animal models. This same approach was used earlier in another mouse model of DS, Tc1. However, due to its manner of creation, roughly 50 of the protein coding genes on HSA21 were non-functional and this model was found to be subject to loss of the human chromosome over development creating mosaic mice that were difficult to study (O’Doherty et al., 2005; Gribble et al., 2013). Improvements in technology have circumnavigated this issue by cloning the 34 Mb q arm of HSA21 into a species specific (mouse or rat) artificial chromosome containing the native centromeric region (Kazuki et al., 2020, 2022). The native centromeric region improves chromosome retention and segregation during mitosis and reduces the mosaicism that confounded the Tc1 model. This strategy has been utilized to develop the latest generation mouse model of DS, MAC21 (Kazuki et al., 2020), as well as the first ever rat model of DS, TsHSA21rat (Kazuki et al., 2022). Each of these models contain 93% of the protein coding genes on HSA21 making it the most complete genetic model to date. These models represent an exciting new opportunity to model DS in an intact system. Early reports suggest that the mouse and rat do recapitulate some DS related phenotypes and more work is eagerly awaited in hopes that these new models will provide insights into the some of the open questions regarding the etiology and development of well characterized DS related phenotypes.

The transchromosomic mouse model, MAC21, has undergone preliminary characterization and recapitulates some of the phenotypes described in individuals with DS (Kazuki et al., 2020). In addition to modeling the genetics of DS, they also show some of the same behavioral deficits in learning and memory. Like Dp1Tyb/Dp(16)1Yey, they appear to recapitulate some of the non-neuronal phenotypes associated with DS such as skeletal changes and cardiovascular abnormalities. Similar to human and Ts65Dn data, in MAC21 the cerebellum is decreased in volume compared to euploid at 4–5 months of age. However, there is no change in hippocampal volume and total brain volume is significantly larger in the trisomic mice. As this is a new mouse model, no characterization of neurodevelopmental changes has been done yet. Assessment of changes in NPC proliferation, neurogenesis, cell and synaptic density will have to be performed to see if this model recapitulates some of the hallmark phenotypes in DS and can be used as an appropriate experimental model.

While there have been dozens of mouse models over the years, the first rat model of DS, TcHSA21rat, was recently created and preliminarily described (Kazuki et al., 2022). While still having the benefits of a rodent model including small size, quick gestation time, and experimental malleability, rats are larger and more social than mice. A rat model of DS creates an exciting opportunity to examine neurological changes and behavior in a more complex model system. Similar to the MAC21 mouse, TcHSA21rat exhibit learning and memory deficits as well as other cardinal features of DS such as craniofacial changes and cardiovascular deficits. Opposed to current mouse models of DS, the TcHSA21rat model recapitulates the decrease in total brain volume that is characteristics in DS. In addition, the cerebellum is specifically decreased in size compared to euploid controls and has a simpler foliation pattern, a feature that is unable to be observed in mouse models. This model was very recently developed and so like the MAC21 model, no neurodevelopmental phenotyping has been performed yet. As this rat model is the first rodent model to capture a key phenotype of DS, the postnatal decrease in brain volume, characterizing changes in neurodevelopment that may be leading to this will hopefully lead to important insight on some of the cellular changes in individuals with DS that are driving the development of the ID.

### Animal model conclusions

Like many other human disorders, rodent models are employed to understand the cellular underpinnings of DS in the hopes of developing future treatments to ameliorate the ID. However, to truly understand the molecular and cellular drivers of different human phenotypes, rodent models must faithfully recapitulate them. While there are many models that mimic both the initial genetics (triplication of HSA21 genes) and ID (deficits in learning and memory), to the best of our knowledge, there are no models that fully recapitulate the known anatomical and cellular changes observed in individuals with DS that are hypothesized to underlie the ID. Ts65Dn historically has recapitulated some of the known phenotypes (volumetric changes during development, neuronal hypocellularity, and changes in proliferation) but lately these phenotypes have been subject to phenotypic drift and unpredictable variability making it difficult to use this model in an effective manner (Shaw et al., 2020). It has also never consistently shown volumetric changes that persist into adulthood except in the cerebellum. Meanwhile Dp1Tyb/Dp(16)1Yey has not shown any of these well described phenotypes even though it models both the genetics and the learning and memory deficits. MAC21, though not well characterized, appears to have an increase in brain volume, the opposite of what is observed in humans. As mentioned, these models do appear to faithfully recapitulate some of the non-neuronal phenotypes and so would be useful in studying those important issues as they also affect quality of life in individuals with DS.

Despite these issues, having an animal model of DS is vitally important. As covered in the next section, induced pluripotent stem cells (iPSCs) are an exciting way to examine molecular and cellular changes in DS but they lack many of the advantages that an animal model can bring to the field. Animals are essential for correlating pharmacological or genetic manipulations with behavioral read-outs, giving context for the effect of the treatments. They are also essential for examining development in an intact system. Cell culture is still unable to fully recapitulate the developmental niches and three-dimensional structure that cells differentiate and migrate in *in vivo*. The TcHSA21rat may provide a solution to the DS modeling issues that continue to plague the mice. Already it appears to model the decrease in brain volume that is an important phenotype in individuals with DS. Hopefully, further characterization will continue to show that this new rodent model captures important features of DS and will provide a next generation platform for study.

## Modeling neurodevelopment with induced pluripotent stem cells

Historically, mouse models have been a cornerstone of the DS research field. They have provided novel and important findings about the genetic and molecular underpinnings of phenotypes due to trisomy 21, as well as provided a platform for preclinical DS research. While animal models have the benefit of studying cellular and molecular underpinnings of DS in an intact tissue system that has behavioral read outs, they lack direct translatability to humans that comes with working with a system that triplicates the full complement of coding and non-coding genes found on HSA21. With the discovery of the “Yamanaka factors,” Oct3/4, Sox2, c-Myc, and Klf4, that are sufficient to reprogram terminally differentiated cells back to a pluripotent state (Takahashi and Yamanaka, 2006), iPSCs derived from individuals with DS have been produced to study cellular and molecular underpinnings of DS while circumnavigating the drawbacks of mouse models. This model system now allows the study of longitudinal cellular differentiation and maturation processes in a human genetic system to identify neurodevelopmental changes that may underlie the ID in DS.

The first iPSCs reprogrammed from an individual with DS were reported in 2008 (Park et al., 2008) and multiple lines have been created from multiple different individuals since then. The current lines are a mix of isogenic and age and sex matched cell lines. The isogenic iPSCs lines consist of paired cell lines that are genetically identical except for the presence or absence of the extra copy of HSA21 in the trisomic and euploid line respectively. In particular, the isogenic pairs are extremely valuable for the field as they eliminate any confounds that may arise from comparing cells with differing genetic backgrounds. It is still not fully understood how different allelic combinations affect the severity of various phenotypes in DS so eliminating that variability is essential for identifying and understanding underlying cellular and molecular neurodevelopmental changes. However, age and sex matched cell sets are also important since the cellular phenotypes that rise above genetic noise elucidate the particular phenotypes that are likely prevalent and impactful in DS. iPSCs derived from people with DS have been differentiated into many types of neuronal and glial cells and are able to recapitulate many of the phenotypes seen in studies of human tissue.

### Neural progenitor cells

One of the best documented phenotypes observed from human studies is the decreased cerebral volume thought to be due in part to hypocellularity driven by a decrease in NPC proliferation (Contestabile et al., 2007; Stagni et al., 2020). Multiple studies have recapitulated this proliferation deficit after differentiating a variety of iPSC cell lines derived from different people with DS into NPCs. In two different isogenic pairs, the trisomic NPCs showed a proliferation deficit compared to their control (Hibaoui et al., 2014; Murray et al., 2015). This consistent phenotype, found in humans, mice, and iPSCs, further supports the hypothesis that a decreased NPC pool leads to the neuronal hypocellularity observed in tissue samples from individuals with DS.

Similar to the mouse models, iPSCs can be used to model aspects of cellular phenotypes in DS that are impossible to study in humans. Recent studies have identified multiple classes of NPCs that result in numerous subtypes of neurons even within a small defined region of the cortex (Tyler et al., 2015; Guillamon-Vivancos et al., 2019). Reports in the Ts65Dn mouse model indicate that NPC subtype may be shifted during development (Tyler and Haydar, 2013). A recent study on an isogenic line of iPSCs differentiated the cells to a ventralized NPC fate and identified a subtype shift with the trisomic progenitors. The trisomic NPCs exhibited more ventral and fewer caudal characteristics compared to their euploid controls exposed to the same patterning agents (Giffin-Rao et al., 2020). This finding was validated in three additional non-isogenic matched trisomic and euploid iPSC lines. A similar study examining changes in the production of interneurons but in an organoid system found an increase in proportion of OLIG2 expressing NPCs in the trisomic organoids differentiated toward a ventral forebrain fate (Xu et al., 2019). This increase in percentage of OLIG2 expressing ventral NPCs was also found in two dimensional culture where two isogenic lines of iPSCs were differentiated to brain-like NPCs and ventralized (Klein et al., 2022). As OLIG2+ NPCs produce both interneurons and oligodendrocytes in the brain, this increase in OLIG2+ cells accompanied by a decrease in *NKX2.2* expression (Klein et al., 2022) could indicate a subtype shift, leading to an increased production of interneurons at the expense of OLs and providing a developmental explanation for some of the observed phenotypes.

### Neurons

In addition to proliferation changes resulting in a smaller progenitor pool, the microencephaly observed in individuals with DS is thought to also be driven by a decrease in neurogenesis. However, using iPSCs to model changes in neurogenesis has produced conflicting results. Two studies using isogenic pairs of iPSCs did not identify any changes in differentiation of excitatory cortical neurons (Weick et al., 2013; Murray et al., 2015). However, two other studies, one using a separate isogenic line and one using non-isogenic matched iPSCs found that the trisomic line produced a decreased number of neurons compared to the euploid control (Lu et al., 2013; Hibaoui et al., 2014).

Interestingly, in a study where age matched trisomic and euploid iPSCs underwent directed differentiation into neurons, there was no significant difference in neuron production between genotypes. However, if the same cell lines were allowed to spontaneously differentiate, the trisomic lines produced a significantly decreased percentage of neurons (Chen et al., 2014). These discordant results reported with the same cell lines perhaps explain the divergent results in previous studies and emphasize the importance of differentiation protocol when examining neurodevelopment in DS. It would be of interest to repeat the spontaneous differentiation protocol with the isogenic lines where no difference in neurogenesis was identified. It may be that the protocols promoted neuronal identity so strongly that they overrode any intrinsic difference in neurodevelopment. If that is the case, using a spontaneous differentiation protocol would better model changes in neurogenesis *in vivo* and would be a more appropriate system to study the molecular drivers of the cellular phenotype.

Similar to NPCs, iPSCs provide the ability to examine neuronal composition in a more detailed manner than tissue studies allow. Of particular interest in the field is understanding the balance of excitatory and inhibitory neurons present in DS. Similar to studies in Ts65Dn, a significant increase in GABAergic interneurons was identified in both isogenic and matched lines of trisomic organoids differentiated to a ventral forebrain fate (Xu et al., 2019). Specifically, an increase in calretinin and somatostatin positive interneurons was identified. However, another study using a 2D culture method identified a significant decrease in calretinin positive interneurons along with a significant decrease in calbindin positive interneurons (Huo et al., 2018). The conflicting results between the above studies may be due to the 3D versus 2D nature of the culture systems or the different cell lines used in the studies. In addition to contradicting each other, neither study precisely matches the human data indicating there is still much work to do, both in understanding the human phenotype and modeling it to understand its molecular origin.

While the nature of any excitatory and inhibitory neurons imbalance is still up for debate, human tissue studies have consistently identified changes in dendritic spine density indicating lifelong alteration of synaptic connections. Recapitulating this finding, a study of iPSC derived early cortical neurons identified a decrease in synapsin positive puncta as well as a decrease in the incidence and frequency of spontaneous post synaptic currents, indicating deficits in the ability to form functional synaptic connections in trisomic neurons (Weick et al., 2013). The decrease in synapsin positive puncta was also reported in trisomic neurons derived from a separate isogenic line (Hibaoui et al., 2014) along with a decrease in number and length of neurites, again recapitulating phenotypes observed in human tissue. Finally in a third study using age matched iPSC lines, trisomic neurons again exhibited significantly decreased neurite length compared to control lines (Chen et al., 2014). A novel study where NPCs and neurons derived from iPSCs were engrafted into mouse cortex and allowed to mature found an increase in spine density in the trisomic neurons compared to euploid controls (Real et al., 2018). However, the human equivalent age of the engrafted neurons (5–8 months) during this time period is comparable to the samples that show an increase spine number in human tissue (Becker et al., 1986). The spines on the trisomic neurons also appeared to be more stable, with less turnover observed in trisomic neurons compared to euploid. Perhaps it is this increased stability that results in the eventual decrease in synapses in trisomic neurons as they are less plastic and unable to adapt with the maturing neurons.

The advantage of any model system is the ability to manipulate and measure phenotypes in ways that are impossible in tissue. Similar to the mouse models, iPSCs have been used to ask questions about functionality that is unable to be studied in fixed human tissue. One study found no change in the electrical properties of either excitatory or inhibitory trisomic early cortical neurons indicating that at that stage, there is no difference in intrinsic ability to communicate (Weick et al., 2013). This finding was repeated using age-matched cell lines that again found no difference in electrical properties between trisomic and euploid neurons (Chen et al., 2014). In the neurons transplanted into the mouse cortex, distinct changes in activity were observed in trisomic neuronal circuitry over time. Fewer trisomic grafts exhibited bursting activity and those that did had reduced bursting frequency (Real et al., 2018). These activity changes indicate that it may be alterations in circuitry rather than changes in intrinsic neuronal properties that may be responsible for altered neuronal activity. However, it is difficult to directly compare activity profiles and electrical properties between different cell lines derived from different individuals and differentiated to distinct maturation states.

### Glia

Alterations in glia have been described in DS in addition to changes in neuronal development. Once of the strongest glial phenotypes observed in iPSC culture is increased astrocyte differentiation at the expense of neuronal differentiation. A significant increase in astrocytes differentiated from trisomic iPSCs has been reported many times (Briggs et al., 2013; Chen et al., 2014; Hibaoui et al., 2014; Real et al., 2018; Mollo et al., 2021). While direct observation of functional changes in astrocytes is not possible in human tissue samples, one of the benefits of using iPSCs as a model is the ability to investigate dynamic cellular processes. Astrocytes differentiated from iPSCs derived from individuals with DS had changes in cell adhesion and motility dynamics compared to matched euploid controls (Bally et al., 2020). While many studies have examined iPSC derived astrocytes, currently, no studies differentiating iPSCs to oligodendrocytes or microglia have been reported in the context of DS though changes in both of these glia types have been reported in humans and mice.

### Induced pluripotent stem cell model conclusions

Induced pluripotent stem cells derived from individuals with DS have allowed the study of longitudinal cellular processes with a human genetic background in a way that has not been possible before due to the limitations of human tissue. Due to this wide array of methodologies and cell lines used to generate a variety of cell types, a range of cellular phenotypes have been observed from the differentiated trisomic cells. It is still unclear whether these differences are due to methodology, genetic background, cell stage, or other currently unknown factors. This variability needs to be carefully considered and work needs to continue to eliminate technical artifacts in culture conditions to allow a fully understanding of the intrinsic neurodevelopmental phenotypes in DS. However, we have still gained a lot of information about a variety of cell types using iPSCs that is unable to be studied from primary tissue samples and is valuable in further our understanding of DS.

## Discussion

Great strides have been made in understanding the neurodevelopmental changes underlying the ID in DS based on detailed studies of human tissue coupled with findings from model systems. Through these combined studies, multiple changes in neural and glial development have been identified. While the field is fortunate to have this immense amount of data, using it as a jumping off point to either elucidate the molecular mechanisms driving the phenotypes or identify pharmacological targets is hampered by the variable and discordant results. To maintain progress, it is essential to reconcile these differences and identify which are the result of technical limitations and which are representative of the underlying variability in DS that we do not yet understand.

**FIGURE 2:**
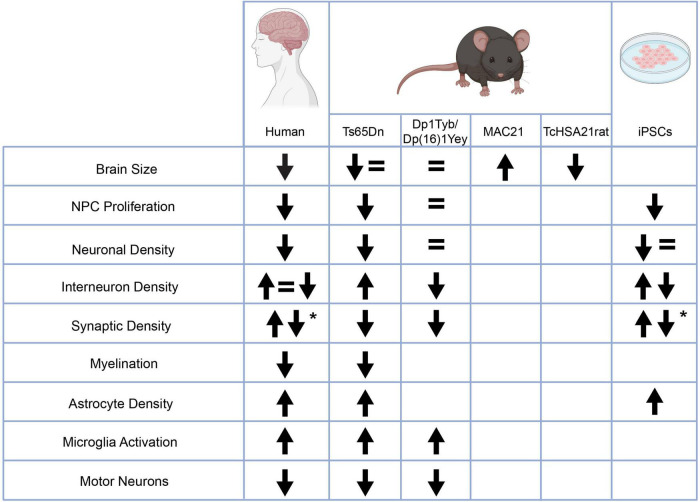
Changes in neurodevelopmental characteristics in people with Down syndrome and various models relative to controls. Some of the observed phenotypes are inconsistent either from human studies or between humans and model systems. Other phenotypes are consistent within human studies and across model systems including an increase in astrocyte density, an increase in microglia activation, a decrease in motor neurons in the spinal cord, and changes in synaptic density. *The up and down arrows in the changes in synaptic density indicate an age dependent phenotype. This figure was created with BioRender.com.

There are some neurodevelopmental phenotypes that have consistently appeared not only in all human tissue studied but also in mouse models and iPSCs. These phenotypes include a decrease in NPC proliferation, changes in synaptic density, and an increase in astrocyte density. Other phenotypes such as a decrease in myelination, an increase in microglia activity, and a decrease in motor neurons in the spinal cord have been consistent between human and mouse studies but have not yet been studied in iPSCs (Figure 2). These phenotypes that arise above the noise of human variability and model system are likely to represent key phenotypes that strongly underlie the ID and now need to be expanded upon. The underlying molecular mechanisms of these phenotypes should be examined to understand their relation to trisomy 21. The consequences of these phenotypes need to be further elucidated as well. For example, is the decrease in NPC proliferation specific to a certain subtype of progenitor cell? Does the decrease in proliferation result particularly affect a neuronal subtype? How does the decrease in synaptic density affect circuit formation? As these phenotypes consistently appear, it is time to move beyond broad strokes into a more nuanced understanding of them.

In contrast to the consistent phenotypes, there are several key phenotypes that are discordant between human studies and model systems. For example, decreased brain volume has been consistently observed in individuals with DS across their lifespan. The phenotype has not been well recapitulated in rodent studies. While this could be attributed to a technical artifact due to the divergence of mice and humans, as this phenotype is not present in the rodent models it is difficult to study the underlying mechanism or elucidate the role the decrease in brain volume plays in the ID in individuals with DS. Hopefully, the decrease in volume observed in the first characterization of the TcHSA21rat will allow this phenotype to be further studied. In a similar vein, neuronal hypocellularity has been consistently described in samples from individuals with DS. However, studies differentiating neurons from iPSCs show conflicting results with some studies reporting no change in neuron differentiation and others reporting decreased neurogenesis in the trisomic cells. As discussed in the iPSC section, it may be that the strength of the differentiation protocol is overriding any intrinsic deficit in the trisomic neurons and more strongly pushing them to their differentiated state than occurs *via* instructive signals *in vivo*. As suggested, an important experiment would be to allow multiple lines of iPSCs to spontaneously differentiate and assess whether a decrease in neurogenesis is observed then. As it is difficult to study the effect that triplication of HSA21 may have on neurogenesis if the phenotype cannot be consistently recapitulated with the current model systems, issues like these that need to be resolved to bring the field into concordance.

Finally, there are other phenotypes that are not consistent within the human tissue studied – specifically changes in interneuron density. Studies have found either an increase, decrease, or no change in interneuron density and mouse and iPSC data is equally contradictory. This phenotype has been a focus of intense study as a change in the proportion of excitatory and inhibitory neurons would exert a large effect on circuit activity and neuronal communication in the brain. Based on data from mouse models, several different clinical trials have tested the effect of GABA antagonists on cognition in people with DS (Hart et al., 2017), however, none of these trials have resulted in an improvement. It is unclear whether this is due to discordance between mouse models and people or due to an unknown biological variable in the individuals studied such as drug administration during a period of neurodevelopment where it is ineffective. It is also possible that this may be a phenotype that is indeed variable in individuals with DS and is highly dependent on each person’s allelic composition. This would explain the divergent results from human tissue samples but would require very careful examination of a broad collection of tissue samples and iPSCs from individuals with DS in order to draw this conclusion with confidence. As there is a wide spectrum of ID in DS, changes in interneuron density may be an interesting candidate for an underlying cellular cause.

The field of neurodevelopment in DS is at an exciting junction. We have a broad understanding of multiple cellular phenotypes that are likely driving the ID in DS. We have multiple model systems we can employ to study these phenotypes. It is now time to bring some of the divergent phenotypes into alignment, or to obtain an understanding of the true phenotypic variability present in DS. Hopefully, the coming decades will result in a deeper understanding of the molecular drivers of the phenotypes and a more subtle understanding of the regional and cell-type effects of trisomy 21. We may even identify some clear pharmacological targets to improve the quality of life of people with DS and their families so they can continue to bring their talents into our communities.

## Author contributions

JK and TH wrote the manuscript. Both authors contributed to the article and approved the submitted version.

## Conflict of interest

The authors declare that the research was conducted in the absence of any commercial or financial relationships that could be construed as a potential conflict of interest.

## Publisher’s note

All claims expressed in this article are solely those of the authors and do not necessarily represent those of their affiliated organizations, or those of the publisher, the editors and the reviewers. Any product that may be evaluated in this article, or claim that may be made by its manufacturer, is not guaranteed or endorsed by the publisher.
